# ﻿Two new species in *Capillidium* (Ancylistaceae, Entomophthorales) from China, with a proposal for a new combination

**DOI:** 10.3897/mycokeys.89.79537

**Published:** 2022-04-29

**Authors:** Yong Nie, Heng Zhao, ZiMin Wang, ZhengYu Zhou, XiaoYong Liu, Bo Huang

**Affiliations:** 1 Anhui Provincial Key Laboratory for Microbial Pest Control, Anhui Agricultural University, Hefei 230036, China Anhui Agricultural University Hefei China; 2 School of Civil Engineering and Architecture, Anhui University of Technology, Ma,anshan 243002, China Anhui University of Technology Ma'anshan China; 3 Institute of Microbiology, School of Ecology and Nature Conservation, Beijing Forestry University, Beijing 100083, China Beijing Forestry University Beijing China; 4 College of Life Sciences, Shandong Normal University, Jinan 250014, China Shandong Normal University Jinan China

**Keywords:** Ancylistaceae, Capilliconidia, morphology, new taxa, phylogeny

## Abstract

A taxonomic revision of *Conidiobolus* s.l. (Ancylistaceae, Entomophthorales) delimited all members that form capilliconidia into the genus *Capillidium*. In this study, we report two new species of *Capillidium* that were isolated in China. *Capillidiummacrocapilliconidium***sp. nov.** is characterised by large capilliconidia. *Capillidiumjiangsuense***sp. nov.** is differentiated by large capilliconidia and long, slender secondary conidiophores. Phylogenetic analyses were performed using sequences from the nuclear large subunit of rDNA (nucLSU), the mitochondrial small subunit of rDNA (mtSSU) and elongation-factor-like (*EFL*). The analyses revealed sister relationships between *Ca.macrocapilliconidium***sp. nov.** and *Ca.globuliferus* / *Ca.pumilum* and between *Ca.jiangsuense***sp. nov.** and *Ca.denaeosporum*. Additionally, a new combination of *Ca.rugosum* (Drechsler) B. Huang & Y. Nie **comb. nov.** is proposed herein. An identification key is provided for the ten accepted *Capillidium* species.

## ﻿Introduction

The taxonomic name *Capillidium* was first introduced as a subgenus within the genus *Conidiobolus* (Ancylistaceae, Entomophthorales) (Ben-Ze’ev and Kenneth 1982). All its members were clustered into a monophyletic group in the family Ancylistacaceae, based on four molecular loci [i.e. small subunit of nuclear rDNA (nucSSU), large subunit of nuclear rDNA (nucLSU), small subunit of mitochondrial rDNA (mtSSU) and elongation-factor-like (*EFL*)] (Nie et al. 2020). Species in this genus are typically characterised by capilliconidia protruding from elongated, slender conidiophores (Nie et al. 2020). Based on this synapomorphy and a re-examination of the protologue for *Conidiobolus* s.l. species (Drechsler 1953a, b, 1954, 1955a, 1957; Srinivasan and Thirumalachar 1967, 1968; Callaghan et al. 2000), seven species so far have been recombined into the monophyletic genus *Capillidium*, including: *Ca.adiaeretum* (Drechsler) B. Huang & Y. Nie, *Ca.bangalorense* (Sriniv. & Thirum.) B. Huang & Y. Nie, *Ca.denaeosporum* (Drechsler) B. Huang & Y. Nie, *Ca.heterosporum* (Drechsler) B. Huang & Y. Nie, *Ca.lobatum* (Sriniv. & Thirum.) B. Huang & Y. Nie, *Ca.pumilum* (Drechsler) B. Huang & Y. Nie and *Ca.rhysosporum* (Drechsler) B. Huang & Y. Nie (Nie et al. 2020).

Although *Capillidium* is a small genus with only seven accepted species, it possesses high morphological diversity. For instance, primary conidia range from 18 μm (*Ca.pumilum*) to 46 μm (*Ca.adiaeretum*) in size (Drechsler 1953a, 1955a); resting spores are present in *Ca.adiaretum*, *Ca.bangalorense* and *Ca.rhysosporum*, but not in *Ca.denaeosporum*, *Ca.heterosporum*, *Ca.lobatum* and *Ca.pumilum* (Drechsler 1953b, 1955a, 1957); *Ca.heterosporum* has slender conidiophores that are branched at the base and end with 2–6 terminal capilliconidia each (Drechsler 1953b), whereas other members are unbranched and end with one capilliconidia (Nie et al. 2020); although nearly all *Capillidium* species only produce capilliconidia, *Ca.adiaeretum* also produces microconidia (Callaghan et al. 2000). These important diagnostic characteristics can help mycologists form a comprehensive understanding of this fungal group.

Two species *Ca.adiaeretum* and *Ca.heterosporum* have been identified in China (Wang et al. 2010; Nie et al. 2020). Continuing investigations into Chinese *Conidiobolus* s.l. led to the discovery of two new species in the genus *Capillidium*. We describe them herein, suggest a new combination for this genus and provide an updated identification key for the species of *Capillidium*.

## ﻿Materials and methods

### ﻿Isolates and morphology

Plant debris was collected from Wanfo Mountain, Shucheng County, Anhui Province, China and Laoshan National Forest Park and Tianwang Town, Jiangsu Province, China. Pre-sterilised plastic bags were used to pack these plant debris samples. Isolation procedures were the same as described by Drechsler (1952) and King (1976a). Plant debris samples were incubated on inverted Petri dishes containing PDA medium (potato 200 g, dextrose 20 g, agar 20 g, H_2_O, 1 litre) at 21 °C for 4 days. The incubated dishes were examined daily under a stereomicroscope (SMZ1500, Nikon Corporation, Japan). When an entomophthoroid fungus appeared, it was transferred to a clean PDA plate for purification and then sub-cultivated for morphological studies. Microscopic structure was observed under a light microscope (BX51, Olympus Corporation, Tokyo, Japan) and imaged using a microscope-camera system (DP25, Olympus Corporation, Tokyo, Japan). The size and shape of the primary conidia, primary conidiophores, secondary conidiophores, capilliconidia etc. were measured and described using the method by King (1976a) and the type of replicative conidia were observed on 2% water agar (agar 2 g, H_2_O, 1 litre). All isolates were deposited at the Research Center for Entomogenous Fungi at Anhui Agricultural University, Anhui Province, China (RCEF) and duplicated at the China General Microbiological Culture Collection Center, Beijing, China (CGMCC). A total of 13 ex-types of *Conidiobolus* s.l. were acquired from the American Type Culture Collection, Manassas, VA, USA (ATCC).

### ﻿DNA extraction, PCR amplification and sequencing

Total cellular DNA was extracted using the method by Watanabe et al. (2010). For phylogenetic analyses, three loci were amplified using relevant primer pairs: LR0R (5’-ACC CGC TGA ACT TAA GC-3’) / LR5 (5’-TCC TGA GGG AAA CTT CG-3’) for nucLSU (Vilgalys and Hester 1990), mtSSU1 (5’-GCW GCA GTG RGG AAT NTT GGR CAA T-3’) / mtSSU2R (5’-GTR GAC TAM TSR GGT ATC TAA TC-3’) for mtSSU (Zoller et al. 1999) and EF983 (5’-GCY CCY GGH CAY CGT GAY TTY AT-3’) / EF1aZ-1R (5’-ACA TCW CCG ACA CCC TTG ATC TTG -3’) for *EFL* (Nie et al. 2012).

PCR amplification was carried out in a 50 µl mixture containing 1 μl dNTPs (200 μM), 1 μl MgCl_2_ (2.5 mM), 10 µl Phusion HF buffer (5×), 1 μl primers each (0.5 μM), 100 ng genomic DNA and 0.5 μl Taq polymerase (0.04 Unit/l, Super Pfx DNA Polymerase, Cowinbioscience Co. Ltd., Shanghai, China). PCR runs were conducted under the following conditions: an initial denaturation step at 94 °C for 3 min followed by 35 cycles of denaturation at 94 °C for 1 min, annealing at 55 / 54 / 57 °C (nucLSU / mtSSU / *EFL*), extension at 72 °C for 1 min; a final extension step at 72 °C for 7 min. DNA sequences were generated on both strands by performing dideoxy-nucleotide chain termination on an ABI 3700 automated sequencer at the Shanghai Genecore Biotechnologies Company (Shanghai, China). Sequences were processed with Geneious 9.0.2 (http://www.geneious.com, Kearse et al. 2012) and deposited in GenBank under the accession numbers listed in Table 1.

**Table 1.** The species used in phylogenetic analyses.

Species	Strains*	GenBank accession numbers			References
		nucLSU	*EFL*	mtSSU	
*Azygosporusmacropapillatus*	CGMCC 3.16068 (T)	MZ542006	MZ555650	MZ542279	Cai et al. (2021)
*A.parvus*	ATCC 14634 (T)	KX752051	KY402207	MK301192	Cai et al. (2021)
*Capillidiumadiaeretum*	ARSEF 451 (T)	KC461182	–	–	GenBank
*Ca.adiaeretum*	CGMCC 3.15888	MN061284	MN061481	MN061287	Nie et al. (2020)
*Ca.bangalorense*	ARSEF 449 (T)	DQ364204	–	DQ364225	Chen and Huang (2018)
*Ca.denaeosporum*	ATCC 12940 (T)	JF816215	JF816228	MK301181	Nie et al. (2012, 2020)
*Ca.globuliferum*	CBS 152.56 (T)	MH869095	–	–	Vu et al. (2019)
*Ca.heterosporum*	CBS 543.63	MH869973	–	–	Vu et al. (2019)
*Ca.heterosporum*	RCEF 4430	JF816225	JF816239	MK301183	Nie et al. (2012, 2020)
*Ca.lobatum*	ATCC 18153 (T)	JF816218	JF816233	MK301187	Nie et al. (2012, 2020)
*Ca.pumilum*	ARSEF 453 (T)	EF392383	–	EF392496	GenBank
*Ca.rhysosporum*	ATCC 12588 (T)	JN131540	JN131546	MK301195	Nie et al. (2018, 2020)
*Ca.rhysosporum*	CBS 141.57	MH869215	–	–	Vu et al. (2019)
*Ca.rugosum*	CBS 158.56 (T)	MH869097	–	–	Vu et al. (2019)
*Ca.marcocapilliconidium*	CGMCC 3.16169 (T)	OL830454	OL801337	OL830457	This article
*Ca.marcocapilliconidium*	RCEF 6332	OL830455	OL801338	OL830458	This article
*Ca.jiangsuense*	CGMCC 3.16168 (T)	OL830456	OL801339	OL830459	This article
*Conidioboluscoronatus*	NRRL 28638	AY546691	DQ275337	–	Lutzoni et al. (2004)
*C.humicolus*	ATCC 28849 (T)	JF816220	JF816231	MK301184	Nie et al. (2012, 2020)
*C.khandalensis*	ATCC 15162 (T)	KX686994	KY402204	MK301185	Nie et al. (2012, 2020)
*C.lichenicolus*	ATCC 16200 (T)	JF816216	JF816232	MK301186	Nie et al. (2012, 2020)
*C.polytocus*	ATCC 12244 (T)	JF816213	JF816227	MK301194	Nie et al. (2012, 2020)
*Microconidiobolusnodosus*	ATCC 16577 (T)	JF816217	JF816235	MK333388	Nie et al. (2012, 2020)
*M.paulus*	ARSEF 450 (T)	KC788409	–	–	Gryganskyi et al. (2013)
*M.terrestris*	ATCC 16198 (T)	KX752050	KY402208	MK301199	Nie et al. (2016, 2020)
*Neoconidioboluscouchii*	ATCC 18152 (T)	JN131538	JN131544	MK301179	Nie et al. (2016, 2020)
*N.mirabilis*	CGMCC 3.17763 (T)	MH282852	MH282853	MK333389	Nie et al. (2018, 2020)
*N.pachyzygosporus*	CGMCC 3.17764 (T)	KP218521	KP218524	MK333390	Nie et al. (2018, 2020)
*N.stromoideus*	ATCC 15430 (T)	JF816219	JF816229	MK301198	Nie et al. (2012, 2020)
*N.thromboides*	ATCC 12587 (T)	JF816214	JF816230	MK301200	Nie et al. (2012, 2020)

*ARSEF, ARS Entomopathogenic Fungus Collection (Ithaca, U.S.A.). ATCC, American Type Culture Collection (Manassas, U.S.A). CBS, Westerdijk Fungal Biodiversity Institute (Utrecht, The Netherlands). CGMCC, China General Microbiological Culture Collection Center (Beijing, China). NRRL, ARS Culture Collection (Peoria, U.S.A). RCEF, Research Center for Entomogenous Fungi (Hefei, China). T = ex-type.

### ﻿Phylogenetic analyses

The data for the three target loci (nucLSU, mtSSU and *EFL*) were produced during this study and during our previous study (Nie et al. 2020). Sequences were retrieved from GenBank and concatenated using SequenceMatrix 1.7.8 (Vaidya et al. 2011). For this analysis, fifteen species in four closely-related genera (*Azygosporus*, *Conidiobolus* s.s., *Neoconidiobolus* and *Microconidiobolus*) served as outgroups (Table 1). Local alignment was conducted with MUSCLE 3.8.31 (Edgar 2004) and manually refined with BioEdit v. 7.2.6 (Hall 1999). The aligned sequence matrix was deposited in TreeBase (https://treebase.org) under the submission ID S29102.

Phylogenetic analyses were performed using three different methods: Maximum Likelihood (ML), Maximum Parsimony (MP) and Bayesian Inference (BI). For ML and BI analyses, best-fit substitution models for each locus were estimated in Modeltest 3.7 using the Akaike Information Criterion (AIC) value (Posada and Crandall 1998). The ML phylogenetic analysis was statistically tested in RAxML 8.1.17 with 1000 bootstrap replicates (Stamatakis 2014). The BI analysis was carried out in MrBayes v.3.1.2 using Markov Chain Monte Carlo (MCMC) methods (Ronquist and Huelsenbeck 2003). Beginning with random starting trees, four MCMC chains ran simultaneously for 1 million generations. The trees were sampled once every 100 generations. These chains stopped when all convergences met and the standard deviation fell below 0.01. MP analyses were conducted using a heuristic search in PAUP* 4.0b10 (Swofford 2002). Bootstrap analyses were conducted with 1000 bootstrap replicates to determine the confidence levels of the nodes within the inferred tree topologies (Felsenstein 1985). Tree bisection-reconnection (TBR) was selected for branch swapping. Phylogenetic trees were checked with FigTree 1.4 (Rambaut 2012) and further modified with iTOL (https://itol.embl.de/).

## ﻿Results

### ﻿Phylogenetic analyses

The concatenated alignment included 30 strains, 15 of which were outgroups from *Azygosporus*, *Conidiobolus* s.s., *Microconidiobolus* and *Neoconidiobolus* (Table 1). The aligned three-locus datasets contained 1861 characters. Amongst these, 852 characters were constant, 159 were parsimony-uninformative and 850 were parsimony informative. The most parsimonious tree had a tree length (TL) consisting of 3445 steps, a consistency index (CI) of 0.5068, a homoplasy index (HI) of 0.4932, a retention index (RI) of 0.7145 and a rescaled consistency index (RC) of 0.3621. The ML and BI analyses were performed using the best models for nucLSU (TrNef+G), *EFL* (TIMef) and mtSSU (K81) partitioning. The final average standard deviation of the split frequencies was 0.0059 and the final likelihood value was -17189. The tree topology from ML analysis was identical to those obtained from MP and BI analyses. The final ML tree was generated with bootstrap support values from MP/ML analyses, as well as posterior probability values from BI analysis at each branch.

**Figure 1.**
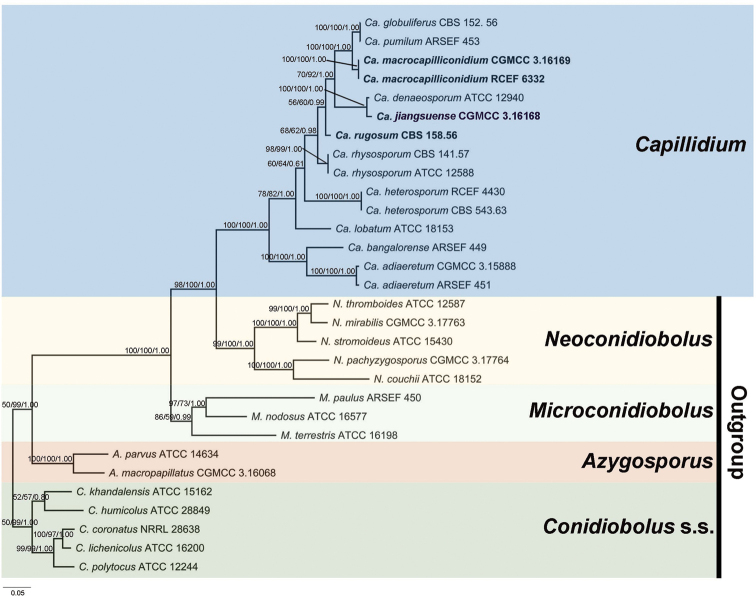
The phylogenetic tree of *Capillidium* constructed using Maximum Likelihood analyses on nucLSU, *EFL* and mtSSU sequences. *Conidiobolus* s.l. species were used as outgroups. New taxa are indicated by bold text. Maximum Parsimony bootstrap values (≥ 50%) / Maximum Likelihood bootstrap values (≥ 50%) / Bayesian posterior probabilities (≥ 0.50) of clades are provided alongside the branches. The scale bar at the lower left indicates substitutions per site.

The phylogeny revealed that three strains belong to the genus *Capillidium*. The strains CGMCC 3.16169 / RCEF 6332 and CGMCC 3016168 were grouped closely with *Ca.pumilum* / *Ca.globuliferus* (100/100/1.00) and *Ca.denaeosporum* (100/100/1.00), respectively.

### ﻿Taxonomy

#### ﻿ Capillidium macrocapilliconidium

Taxon classificationFungiEntomophthoralesAncylistaceae

B. Huang & Y. Nie sp. nov.

9433C5CA-C055-5CE6-A061-0EF291C0CDC4

842227

Fig. 2

##### Etymology.

*macrocapilliconidium* (Lat.), referring to the large size of its capilliconidia.

##### Known distribution.

Jiangsu Province, China.

##### Typification.

China, Jiangsu Province, Nanjing City, Laoshan National Forest Park, 32°5'52"N, 118°35'37"E, from plant debris, 1 Dec 2018, *Y. Nie and Y. Gao*, culture ex-holotype *CGMCC 3.16169 (=RCEF 6553)*.

##### Additional specimens examined.

China, Anhui Province, Shucheng County, Wanfo Mountain, 31°9'51"N, 116°57'86"E, from plant debris, 13 Mar 2016, X.X. Tang, culture RCEF 6332. GenBank: nrLSU = OL830455; *EFL* = OL801338; mtSSU = OL830458.

##### Description.

Colonies on PDA at 21 °C after 3 d white, reaching ca. 28 mm in diameter, yellowish after 10 d. Mycelia hyaline, 5.5–10 μm wide, often branched. Primary conidiophores arising from hyphal segments, hyaline, 70–250 × 5–13 μm, unbranched and producing a single globose primary conidium, widening upwards near the tip. Primary conidia forcibly discharged, globose to subglobose, 25–34 × 20–28 μm, papillate or conical, 7–10 μm wide, 3–8 μm long. Secondary conidiophores short or long, arising from primary conidia, bearing a single replicative conidium similar to, but smaller than those primary ones and forcibly discharged, producing another kind of replicative conidia called capillidiconidia from slender secondary conidiophores on the 2% water agar. Capillidiconidia colourless, elongate ellipsoidal, 25–37 μm long, 14–17 μm wide. Slender secondary conidiophores unbranched, 85–130 μm long, 4–6 μm wide at the base, tapering gradually to a width of 1–2 μm at the tip. Zygospores usually formed between adjacent segments of the same hypha after 10 d, yellowish, mostly boldly wrinkled, sometimes smooth, globose, elongate ellipsoidal or irregular, 18–35 μm long, 17–28 μm wide, with a wall 1–2 μm thick.

**Figure 2.**
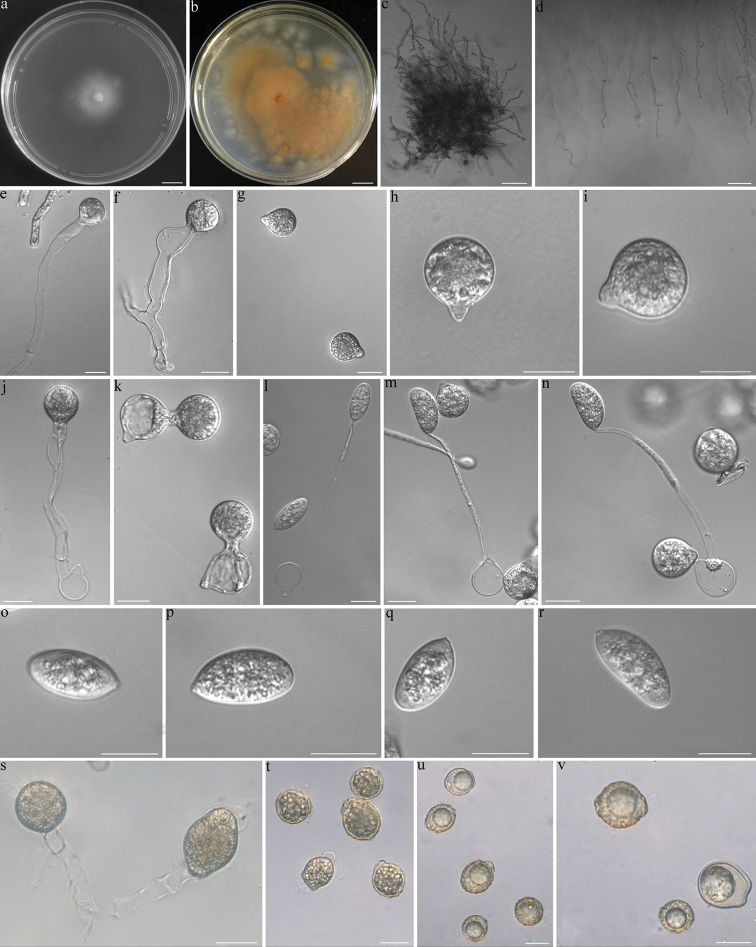
*Capillidiummacrocapilliconidium***a** colony on PDA after 3 d at 21 °C **b** colony on PDA after 10 d at 21 °C **c** Mycelia **d** Mycelia unbranched at the edge of the colony **e, f** primary conidiophores bearing primary conidia **g, h, i** primary conidia **j, k** primary conidia bearing a single secondary conidium **i, m, n** a primary conidium bearing a single capilliconidium **o, p, q, r** Capilliconidia **s** zygospores that were formed on adjacent segments of the same hypha **t** immature zygospores **u, v** mature zygospores. Scale bars: 10 mm (**a–b**); 100 μm (**c–d**); 20 μm (**e–v**).

##### Notes.

*Capillidiummacrocapilliconidium* is characterised by having larger capilliconidia compared to other *Capillidium* species. It produces yellowish and wrinkled zygospores like *Ca.rhysosporum* (Drechsler 1954). However, *Ca.macrocapilliconidium* has larger capilliconidia than *Ca.rhysosporum* (25–37 × 14–17 μm in *Ca.macrocapilliconidium* vs. 12–32 × 6.5–16 μm in *Ca.rhysosporum*). *Ca.macrocapilliconidium* is phylogenetically distant from *Ca.rhysosporum* (Fig. 1) and most closely related to *Ca.pumilum*. It is distinguished from *Ca.pumilum* by larger primary conidia (25–34 × 20–28 μm in *Ca.macrocapilliconidium* vs. 9–18 × 7.3–14 μm in *Ca.pumilum*) and capilliconidia (25–37 × 14–17 μm in *Ca.macrocapilliconidium* vs. 8.8–12 × 5–7.5 μm in *Ca.pumilum*) (Drechsler 1955b).

#### ﻿ Capillidium jiangsuense

Taxon classificationFungiEntomophthoralesAncylistaceae

B. Huang & Y. Nie sp. nov.

FEEECB5A-1DCC-5FA1-9DFF-C083E5561569

842228

Fig. 3

##### Etymology.

*jiangsuense* (Lat.), referring to the region where the fungus was isolated.

##### Known distribution.

Jiangsu Province, China.

##### Typification.

China, Jiangsu Province, Jurong City, Tianwang Town, 31°6'94"N, 119°26'91"E, from plant debris, 25 Mar 2018, *Y. Nie*, culture ex-holotype *CGMCC 3.16168 (=RCEF 6545)*.

##### Description.

Colonies on PDA at 21 °C after 3 d white, reaching ca. 21 mm in diameter. Mycelia haline, often unbranched, vegetative hyphae filamentous, 5–10 μm wide. Primary conidiophores unbranched, producing a single primary conidium, widening upwards near the tip, 50–240 × 6–10 μm. Primary conidia forcibly discharged, subglobose to turbinate, 21–31 × 12–29 μm. Papilla 4–10 μm wide, 2–4 μm long. Replicative conidia two kinds on 2% water agar, arising from primary conidia, one similar and smaller to the primary conidia, the other elongate and passively detached, 17–32 × 10–15 μm. Slender secondary conidiophores unbranched, 65–120 μm long, 2.5–3 μm wide at the base, tapering gradually to a width of 1 μm at the tip. Resting spore not observed.

**Figure 3.**
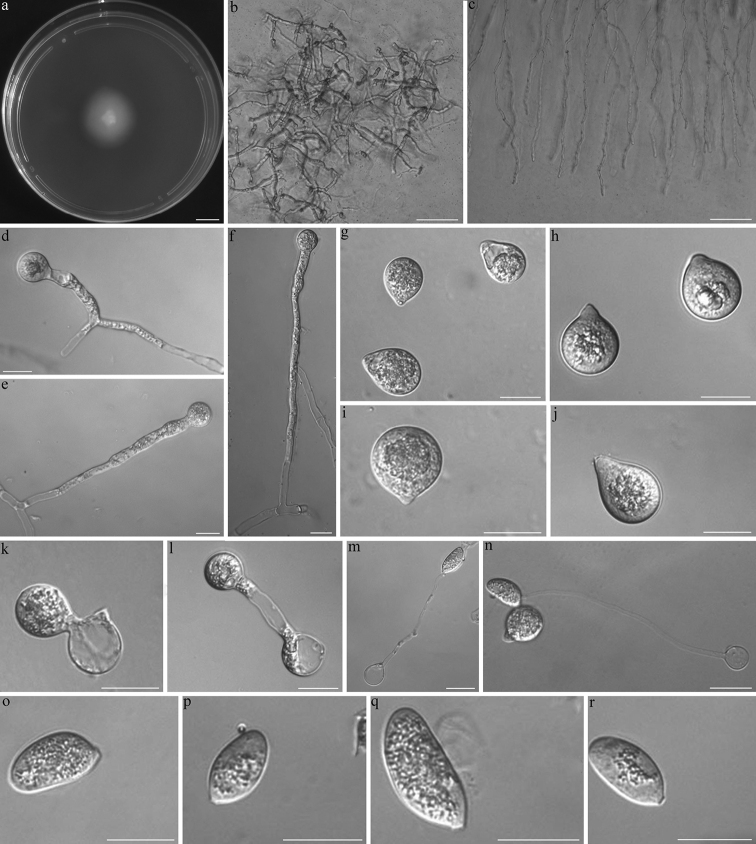
*Capillidiumjiangsuense***a** colony on PDA after 3 d at 21 °C **b** Mycelia **c** Mycelia unbranched at the edge of the colony **d, e, f** primary conidiophores arising from mycelia segments **g, h, i, j** primary conidia **k, i** secondary conidia arising from primary conidia **m, n** primary conidia bearing a single capilliconidium **o, p, q, r** Capilliconidia. Scale bars: 10 mm (**a**); 100 μm (**b, c**); 20 μm (**d–r**).

##### Notes.

Morphologically, the present isolate resembles *Ca.denaeosporum* because of the size of its primary conidia (13–32 × 6–21 μm in *Ca.denaeosporum* vs. 21–31 × 12–29 μm in *Ca.jiangsuense*) (Drechsler 1957). However, *Ca.denaeosporum* has larger capilliconidia (10–18 × 6–10 μm in *Ca.denaeosporum* vs. 17–32 × 10–15 μm in *Ca.jiangsuense*) and longer, more slender secondary conidiophores (35–65 μm in *Ca.denaeosporum* vs. 65–120 μm in *Ca.jiangsuense*) (Drechsler 1957). Although they grouped together with relatively little divergence on the phylogram, DNA similarity levels between the two species are only around 97.9% (nucLSU) (Nie et al. 2012). This evidence supports the present isolate being a new species, which we have named *Capillidiumjiangsuense* sp. nov.

#### ﻿ Capillidium rugosum

Taxon classificationFungiEntomophthoralesAncylistaceae

(Drechsler) B. Huang & Y. Nie comb. nov.

3D839536-F9ED-520C-8BB6-F183B93B704D

842229

##### Basionym.

*Conidiobolusrugosus* Drechsler, Am. J. Bot. 42: 437 (1955).

##### Description.

Refer to Drechsler (1955a).

##### Notes.

The ex-type living culture is ATCC 12586 (United States, New Jersey, Moorestown, 25 February 1954, Drechsler). Historically, *Conidiobolusrugosus* was synonymised with *Co.heterosporus* (King 1976b). However, we have re-established its taxonomic status at the species level, based on the phylogeny herein and the morphological traits of the capilliconidia.

## ﻿Discussion

From the 1950s-1970s, a total of eight *Conidiobolus* species have been reported to produce capilliconidia, including *Conidiobolusdenaeosporus*, *Co.globuliferus*, *Co.heterosporus*, *Co.inordinatus*, *Co.lobatus*, *Co.pumilus*, *Co.rhysosporus* and *Co.rugosus* (Drechsler 1953a, b, 1954, 1955a, b, 1956, 1957; Srinivasan and Thirumalachar 1968). Based on the numerical taxonomy of *Conidiobolus* (King 1976a, b, 1977), four species were rejected. *Co.rugosus* was considered synonymous with *C.heterosporus*. On the other hand, *Conidiobolusdenaeosporus*, *Co.globuliferus* and *Co.inordinatus* were considered synonymous with *Co.pumilus*. Consequently, only four species forming capilliconidia were accepted into this genus. Based on this synapomorphy, the subgenusCapillidium was erected in the latter taxonomic study of *Conidiobolus* (Ben-Ze’ev and Kenneth 1982; Humber 1989). Interestingly, it appears that *Co.adiaeretus* and *Co.bangalorensis* develop both microconidia and capilliconidia (Callaghan et al. 2000). Unfortunately, there was no molecular evidence at the time to support these morphological results. Recently, we summarised molecular data from available *Conidiobolus* s.l. ex-types and identified a monophyletic lineage of *Capillidium* producing capilliconidia. Since then, some taxonomic revisions have been conducted. For example, *Co.denaeosporus* was separated from *Co.pumilus* and recombined into *Capillidium*. *Co.adiaeretus* and *Co.bangalorensis* were also recombined into *Capillidium*. In total, *Capillidium* now has seven accepted species.

*Conidiobolusheterosporus* (= *Capillidiumheterosporum*) and *Co.rugosus* share distinct morphological characteristics. For instance, *Co.heterosposus* bears no resting spores and has conidiophores that are often branched at the base and bear 2–6 terminal capilliconidia (Drechsler 1953a). The conidiophores of *Co.rugosus*, though, have yellowish zygospores with wrinkled or smooth surfaces, are unbranched and bear a single capilllicondiuma (Drechsler 1955b).

Based on a phylogenetic analysis of three gene regions (nucLSU, mtSSU and *EFL*), the ex-type of *Co.rugosus* (Strain No: CBS 158.56) and *Co.heterosposus* diverged into two distinct lineages. Consequently, we identified *Co.rugosus* as an independent species and recombined it into *Capillidium* as a new combination: *Capillidiumrugosum* (Drechsler) B. Huang & Y. Nie comb. nov. On a side note, while researchers previously considered *Co.denaeosporus* (= *Ca.denaeosporum*), *Co.globuliferus* and *Co.inordinatus* to be synonymous with *Co.pumilus* (= *Ca.pumilum*) (King 1976b), the present phylogeny confirmed that *Co.denaeosporus* (= *Ca.denaeosporum*) is an independent species and *Co.globuliferus* is synonymous with *Co.pumilus* (= *Ca.pumilum*). More molecular evidence is needed to clarify the taxonomic status of *Co.inordinatus*.

*Capillidiumbangalorense* may be another *Capillidium* species that forms microspores, based on its close phylogenetic relationship with *Ca.adiaeretum*. Besides microspores, these two species possess another morphological characteristic that is distinctive compared with the other members of *Capillidium*, that being the width between the primary conidiophores and the hyphae (Drechsler 1955a; Srinivasan and Thirumalachar 1967). This could explain why *Ca.adiaeretum* and *Ca.bangalorense* are grouped into a single clade in the phylogenetic tree (Fig. 1). However, *Ca.bangalorense* should be re-examined and more evidence should be supplied to confirm that this clade is in a separate taxon.

With the current description of *Azygosporus*, most members of *Conidiobolus* s.l. have now received suitable taxonomic placements. Yet, there are still many other taxonomic challenges to be resolved in the future, such as replacing the missing ex-type *Co.utriculosis* and assigning *Co.coronatus* as the epitype of *Conidiobolus* s.s., isolating lost ex-types to confirm their taxonomic placements etc. (Nie et al. 2018, 2020, 2021; Cai et al. 2021). For the first time, this study used partial sequence data from nucLSU, mtSSU and *EFL* genes to identify two new species of *Capillidium* from China, increasing the total number of species in the genus to ten. A key to the species of *Capillidium* is provided below.

### ﻿Key to the Species of *Capillidium*

**Table:** 

1	Capilliconidia and microconidia produced, the width of primary conidiophores offers a pronounced dimensional contrast with the mycelial filaments	**2**
–	Only capilliconidia produced, the width of primary conidiophores offers a similar dimensional contrast with the mycelial	**3**
2	Primary conidia larger, up to 46 μm, chlamydospores produced	***Ca.adiaeretum***
–	Primary conidia smaller, less than 25 μm, zygospores produced	***Ca.bangalorense***
3	Slender conidiophores branched at the base, bearing 2–6 terminal capilliconidia	***Ca.heterosporum***
–	Slender conidiophores unbranched at the base, bearing a single capilliconidia	**4**
4	Resting spores of zygospores produced, yellowish, mostly wrinkled, sometimes smooth	**5**
–	Resting spores not observed	**6**
5	Primary conidia and zygospores larger, more than 30 μm	**7**
–	Primary conidia and zygospores smaller, less than 25 μm	***Ca.rugosum* comb. nov.**
6	Primary conidia larger, more than 30 μm	**8**
–	Primary conidia smaller, less than 26 μm	**9**
7	Capilliconidia larger, up to 37 μm	***Ca.macrocapilliconidium* sp. nov.**
–	Capilliconidia smaller, less than 32 μm	***Ca.rhysosporum***
8	Capilliconidia larger, 17–32 × 10–15 μm, primary conidiophores longer, 50–240 μm	***Ca.jiangsuense* sp. nov.**
–	Capilliconidia smaller, 10–18 × 6–10 μm, primary conidiophores shorter, 15–50 μm	***Ca.denaeosporum***
9	Primary conidia larger, 21–26 × 20–24 μm, capilliconidia larger, 18–25 × 8–10 μm	***Ca.lobatum***
–	Primary conidia smaller, 9–18 × 7.3–14 μm, capilliconidia smaller, 8.8–12 × 5–7.5 μm	***Ca.pumilum***

## Supplementary Material

XML Treatment for Capillidium macrocapilliconidium

XML Treatment for Capillidium jiangsuense

XML Treatment for Capillidium rugosum
